# An Investigation into the Impact of Pre-Adolescent Training on Canine Behavior

**DOI:** 10.3390/ani11051298

**Published:** 2021-04-30

**Authors:** Ian R. Dinwoodie, Vivian Zottola, Nicholas H. Dodman

**Affiliations:** 1 Cummings School of Veterinary Medicine, Tufts University, North Grafton, MA 01536, USA; ian.dinwoodie@tufts.edu; 2 Center for Canine Behavior Studies, Salisbury, CT 06068, USA; 3 Boston K9 Concierge LLC, Boston, MA 02127, USA; vivian@dogbehaviorandconsulting.com

**Keywords:** dogs, survey, questionnaire, behavior problems, training

## Abstract

**Simple Summary:**

Current thinking about puppy training is that it should be performed as early in a dog’s life as possible to prevent the later development of behavior problems. However, no study has been performed to see if early puppy training (before 3 months of age) does present clear advantages over training at a later age, in terms of the subsequent development of adult behavior problems. This retrospective study examined the age at which adult dogs were trained as puppies and whether there were advantages of training puppies before 4 months of age or between 5 and 6 months of age. We found no difference in the age of puppy training and the subsequent development of behavior problems. Aggression, compulsive behavior, destructive behavior, and excessive barking were all reduced in dogs that had attended puppy training before 6 months of age compared to a control group of dogs that had not attended puppy training classes. Ancillary findings about the entire study population were that dogs acquired as pups at 12 weeks of age or less had reduced odds of exhibiting fear or anxiety and engaging in destructive behavior. In addition, male dogs were found to have reduced odds of developing aggressive behavior, compulsive behavior, and mounting/humping and increased odds of rolling in repulsive materials. Neutered dogs of either sex were found to have increased odds of developing fear and anxiety, increased odds of escaping/running away, exhibiting coprophagia, and rolling in repulsive materials. The odds of problematic jumping decreased with age.

**Abstract:**

An online survey about puppy training was sent to members of the Center for Canine Behavior Studies and posted on our social media platforms. Six hundred forty-one (641) qualifying owners provided information on 1023 dogs. About half (48%) of the dogs involved in the study attended puppy training and the balance (52%) did not. The goal of the study was to find out whether puppy training at various ages (1–3 months, 4 months, 5–6 months) helped prevent behavior problems later in life (≥1 year). Attending training at 6 months of age or younger resulted in 0.71 the odds of developing aggressive behavior (95% CI: 0.53–0.97; *p* = 0.030), 0.64 the odds of having a compulsive behavior (95% CI: 0.45–0.92; *p* = 0.015), 0.60 the odds of exhibiting destructive behavior (95% CI: 0.37–0.96; *p* = 0.035), 0.68 the odds of excessive barking (95% CI: 0.47–0.99; *p* = 0.043), and 1.56 the odds of house soiling (95% CI: 1.08–2.27; *p* = 0.019). Ancillary findings about the entire study population were that dogs acquired at 12 weeks of age or younger were found to have 0.65 the odds of fear/anxiety (95% CI: 0.46–0.92; *p* = 0.016) and 0.50 the odds of exhibiting destructive behavior (95% CI: 0.31–0.79; *p* = 0.003). In addition, male dogs were found to have 0.68 the odds of developing aggressive behavior (95% CI: 0.53–0.88; *p* = 0.003), 0.66 the odds of developing compulsive behavior (95% CI: 0.49–0.88; *p* = 0.006), 0.37 the odds of mounting/humping (95% CI: 0.26–0.52; *p* < 0.001), and 1.53 the odds of rolling in repulsive materials (95% CI: 1.18–1.97; *p* = 0.001). Neutered dogs of either sex were found to have 3.10 the odds of fear/anxiety (95% CI: 2.05–4.72; *p* < 0.001), 1.97 the odds of escaping/running away (95% CI: 1.12–3.69; *p* = 0.025), 2.01 the odds of exhibiting coprophagia (95% CI 1.30–3.19; *p* = 0.002), and 1.72 the odds of rolling in repulsive materials (95% CI: 1.12–2.66; *p* = 0.014). The odds of problematic jumping deceased by 0.84 for each 1-year increase in age (95% CI: 0.80–0.88; *p* < 0.001).

## 1. Introduction

Prediction of an adult dog’s social behavior relies on many factors stemming from the intersections of heredity and the external environment [1]. The learning process further influences behavior through trial and error [2]. Dog owners intervene proactively in this process by enlisting the help of dog trainers to teach them how to reinforce desired behaviors and suppress unwanted behaviors through antecedent management and structured lessons [3,4]. Most agree that dog owners (guardians) who learn and employ best practices rooted in reward-based training for managing and preventing undesired behaviors in dogs establish a more trusting and prolonged relationship [5,6,7,8].

The ideal period to acquire a young dog, whether through breeder or rescue, is at 8 weeks of age when bonding and emotional attachment to humans are known to occur [1,9]. Novel exposure to various stimuli is also critical during this phase of development. The primary socialization period is finite, supposedly terminating at 3 months of age. However, a secondary period of rapid learning may occur up to 6 months of age and possibly even up to 9 months of age [10].

Of the five canine life stages, the first few months encompassing the neonatal, transitional, and socialization periods (0–12 weeks) are thought to be most influential for early brain development and long-term social and behavioral resiliency [1]. Lack of exposure to stimuli during this neurodevelopment period can result in a lifetime of neophobia and poor decision making [1,11]. In addition, limited early exposure to animate and inanimate cues results in fear and avoidance of environmental stimuli, which may affect future learning and, consequently, behavioral outcomes [9,12]. 

According to the American Veterinary Society of Animal Behavior (AVSAB) position statement, puppy socialization should start as early as 7 or 8 weeks, and structured training classes before 3 months of age [13]. A study of dogs living in urban environments with no training between 7 and 16 weeks were more likely to be fearful as adults [14]. However, there is a paucity of evidence that training and socialization of puppies in the first 6 months of life is effective in reducing behavior problems [10]. In a relatively small retrospective study, Gonzalez-Martinez [4] found that puppies and juveniles that had attended classes had more favorable scores for family-dog aggression, trainability, nonsocial fear, and touch sensitivity. That study did not distinguish the behavioral consequences of early (<3 months) and later (3–6 months) puppy training and only positive (reward-based) training was employed. Other studies found puppy training at under 3–4 months of age caused a reduction in fearful responses toward strangers in adult dogs that had attended classes as puppies [15,16]. The rationale for the timing of puppy training appeared to be that it coincided with the sensitive period of learning [17].

Based on a litany of evidence in animal (and human) literature, dog training, regardless of age, should be free of fear, pain, and intimidation allowing the learner freedom to make mistakes (trial-and-error learning) without fear of retribution, which interferes with long-term learning [5,6,18,19]. Application of positive training methods, including reward-based operant conditioning, counterconditioning, desensitization, shaping, and luring, has proven effective in improving learning and compliance, lowering distress, and reducing long-term conflict between humans and animals. Training that relies on clear communication, establishing operations, and managing expectations whereby the learner is informed when he/she problem solves correctly has shown to increase the frequency of wanted behaviors for dog owners and lower frustration in dogs [5,7,10]. On the other hand, training methods based on positive punishment and negative reinforcement are related to higher incidences of behavior problems, aggression, and fear and in some studies have been shown to increase stress hormones [7,9]. 

Living as companions with humans in a small inter-species social group, dogs become enculturated to the human lifestyle. Early exposure and socialization with other species, including humans and other pet companions, play an important role in helping to shape well-adjusted adult dogs and acceptable societal behaviors. Structured puppy lessons and unstructured puppy play engagements provide learning opportunities for young dogs. Arguably both are important and the biggest difference between the two types of social learning engagements is one focuses on structured communication with humans and the other focuses on communication with other conspecifics. When comparing the behavioral effects of structured puppy training with humans to puppy socialization alone, researchers found puppies that attended structured training scored better on “command responses” than those that attended only socialization engagements or “puppy parties” [15,20]. Structured puppy training classes in young dogs have been shown to reduce the risk of dogs’ aggression to unfamiliar people [16,20]. On the other hand, it has been shown that dogs attending either puppy socialization/training classes or puppy parties/socialization groups prior to 6 months of age had significantly lower total problem behavior scores [10]. Researchers suggest that puppy training classes with qualified professionals help to identify problematic behaviors in young dogs early, thereby allowing interventions to improve behaviors [8,21].

The present study sought to explore the optimal age for puppy training, whether training in the first 3 months produces better outcomes than the ensuing months and confirm the optimal training methods and techniques employed.

## 2. Materials and Methods

### 2.1. Data Collection

The questionnaire for this study was developed via group consensus by a panel of subject matter professionals and experts in the field. For electronic data capture, the questionnaire was hosted online using Typeform, an online survey service platform. A link to the public questionnaire was posted on social media platforms (Twitter, Instagram, and Facebook) and distributed to members of the Center for Canine Behaviors Studies via email. Data collection spanned 6 weeks starting from the first day of September 2019. The study was open to all dog owners and the questionnaire was designed to collect information about a single dog. Dog owners with more than one dog were prompted to fill out the questionnaire for each of their dogs. Email addresses were converted to random MD5 checksums (i.e., 128-bit hashes) and then discarded by the survey platform. These hash values were used to group individual dog responses by household. No identifying information for either a dog or its owner was present in the raw data set. A logic-annotated copy of the questionnaire for the study is available in the Supplementary File S1.

In this retrospective study, the control group consisted of dogs that had not attended puppy training in the first 6 months of life and the treatment group consisted of those that had. Our inclusion criteria consisted of an age range; dogs aged from 1 to 35 years old, inclusive. The upper limit of the inclusion criteria was chosen to account for the possibility that there may exist a dog older than the verified longest living dog without exceeding the bounds of reason.

Logically, the distributed questionnaire could be considered in three parts: (1) questions about a single dog, (2) question about pre-adolescent training, and (3) questions about the presentation of behavior problems. Questions about the dog included acquisition age, age at the time of the study, sex, and neuter status. Questions about pre-adolescent training consisted of the number of classes attended, age of attendance, training technique utilized (e.g., reward-based), and restraining devices employed (e.g., nylon slip collar). The behavior problems under investigation consisted of (1) aggression, (2) compulsion, (3) coprophagia, (4) destruction, (5) escaping/running away, (6) excessive barking, (7) fear/anxiety, (8) house soiling, (9) hyperactivity/overactivity, (10) mounting/humping, (11) problematic jumping, and (12) rolling in repulsive materials (e.g., feces). For each behavior problem, owners were provided with a checklist of common presentations (e.g., aggression toward familiar dogs at home, aggression toward veterinarians) and were asked to indicate which, if any, were applicable to their dog. Such questions generally took the form of “[i]s there at least one situation in which [your dog] behaves [problematic behavior (e.g., aggressively)]?” House soiling was an exception where the question took the form “[h]ave you ever had a problem with [your dog] soiling in the house?” The wording was intended to help avoid false-positive responses for non-problematic house soiling incidents that occurred during house training. The behavioral questions were treated as dichotomous; a non-zero count of presentations indicated the presence of the behavior in question.

### 2.2. Descriptive Analysis

The study data set was exported from Typeform as a comma-separated values (CSV) file. All analyses were performed using the R programming language [22] provided by the R Foundation for Statistical Computing. Descriptive statistics were calculated. Ranges were provided for all medians. Counts were provided for all proportions for clarity.

### 2.3. Analytical Modeling

Probabilities of puppy training attendance were calculated for each year of age and the resulting data were fit with a single linear regression model to assess the relationship between dog age in years and the likelihood of attendance. Outlying points with high leverage, as indicated by a large Cook’s distance, were excluded from the regression. The confidence interval (CI) was calculated using the profile likelihood function without assumption of normality. The significance level was set to α ≤ 0.05 for all regression models in this manuscript.

Binary logistic regression models were built to assess the relationship between attending pre-adolescent training (true/false) and the occurrence of a specific behavior problem (true/false) when the effects of confounding or intervening background variables were accounted for. Separate models were constructed for each of the aforementioned behavior problems (i.e., 12 models were built). Background variables consisted of (1) whether or not the dog had been acquired at 12 weeks old or younger (true/false) as well as (2) dog sex (male/female), (3) neuter status (true/false), and (4) age at the time of the study. In all cases, the null hypothesis represented the statistically independent outcome. Odds ratios (ORs) were calculated as a measure of effect size. Confidence intervals were calculated using the profile likelihood function without assumption of normality. Multicollinearity was assessed using the variance inflation factor (VIF).

Additional binary logistic regression models were built to determine the relationship between variable factors of puppy training for those who had attended and the occurrence of a specific behavior problem (true/false) when the effects of confounding or intervening background variables were accounted for. The variable factors of puppy training we investigated consisted of (1) the starting age of attendance (≤3, 4, or 5–6 months), (2) number of classes attended (1–3, 4–6, 7–9, or 10+ classes), (3) type of training employed (reward-based/punishment-based), and (4) the use of restraining devices (one or more of: buckle collars, metal choke collar, prong, shock, nylon slip collar, harness, head halter, or martingale). The set of controlled background variables matched those mentioned previously and, similarly, separate models were built for each of the behavior problems (i.e., an additional 12 models were built). Effect size, confidence intervals, and multicollinearity were calculated and reported just the same. Per the default behavior of the generalized linear models produced by R’s stats package, entries with missing values for independent variables (e.g., no provided training starting age or number of attended classes) were automatically excluded from the models.

## 3. Results

### 3.1. Demographics

Responses for 1095 dogs were submitted by 669 dog owners. After application of our inclusion criteria, responses for 1023 dogs from 641 dog owners remained. The median number of dog responses submitted per household was one dog response (range: 1 to 8 dog responses per household). The median age of the dogs was 7 years (range: 1 to 19 years). Forty-nine percent (*n* = 497) of the dogs in the study were males, 89% (*n* = 441) of which were castrated; 51% (*n* = 526) were females, 86% (*n* = 450) of which were spayed. In total, 87% (*n* = 891) of dogs in the study were neutered.

Ninety-nine percent (*n* = 1016) of dogs in the study were reported to have exhibited at least one type of behavior problem. The prevalence of the investigated behavior problems were as follows: 78% (*n* = 798) house soiling, 70% (*n* = 713) fear/anxiety, 54% (*n* = 549) aggression, 42% (*n* = 428) rolling in repulsive materials, 37% (*n* = 381) coprophagia, 25% (*n* = 254) compulsion, 22% (*n* = 230) escaping/running away, 22% (*n* = 230) problematic jumping, 21% (*n* = 217) excessive barking, 19% (*n* = 190) mounting/humping, 13 % (*n* = 131) destruction, and 11% (*n* = 116) hyperactive/overactive.

Forty-eight percent (*n* = 494) of the dogs attended training sessions at an age of 6 months old or younger. The balance (*n* = 529) did not attend puppy training and constituted the control group for the study. Forty-seven percent (*n* = 234) of the dogs that attended puppy training started in the 1–3-month range, 26% (*n* = 130) at 4 months, 24% (*n* = 118) in the 5–6-month range, and the balance (*n* = 12) did not provide information on a specific starting age. Forty-four percent (*n* = 215) of the dogs that attended puppy training did so at or before 4 months of age (defined by us as early-age puppy training) and 46% (*n* = 226) percent attended during the 5–6-month range. Ten percent (*n* = 49) of the dogs that attended puppy training attended 1–3 training sessions, 24% (*n* = 120) attended 4–6 sessions, 15% (*n* = 72) percent attended 7–9 sessions, 49% (*n* = 242) attended 10 or more sessions, and 2% (*n* = 11) did not provide information on the number of sessions attended. Eighty-nine percent (*n* = 440) of dogs were reported to have been trained with reward-based training and the balance (*n* = 54) were involved in a training program that involved some form of punishment (including those trained with a “tough love” training style). A restraining device was used for 87% (*n* = 432) of the dogs that attended early-age training. The most common restraining devices were buckle collars, which were employed for 48% (*n* = 235) of the dogs, followed by harnesses for 30% (*n* = 149), martingales for 18% (*n* = 90), nylon slip collars for 9% (*n* = 45), prong collars for 6% (*n* = 33), metal choke collars for 5% (*n* = 27), head halters for 5 % (*n* = 26), and shock collars for 2% (*n* = 9) of the dogs. A punishing restraining device (i.e., a martingale, slip, choke, prong, or shock collar) was employed for 28% (*n* = 124) of all the dogs that were reported to have been trained with a reward-based program. In total, 36% (*n* = 178) of dogs that attended puppy training were subjected to at least one type of punishing restraining device.

### 3.2. Age and Attendance

**Figure 1:**
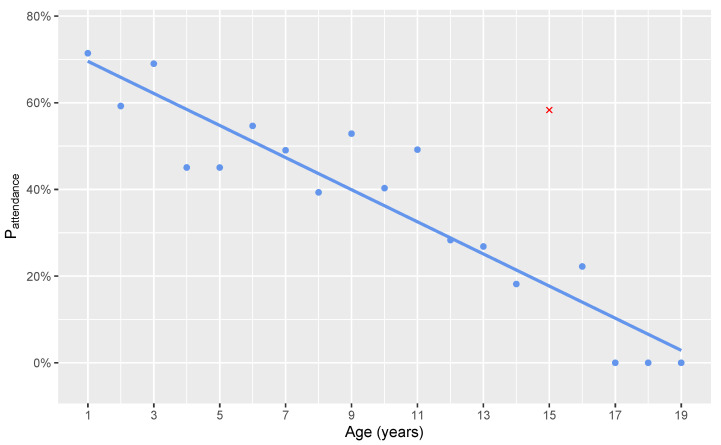
A smooth geometric means plot for the probability of training attendance at 6 months of age or younger versus the current age of the dog in years. The sole outlier has been marked in red to indicate its exclusion from the model.

A single linear regression was calculated to predict the probability of attending puppy training classes based on dog age at the time of the study. A significant association was found (F(1, 16) = 108.3, *p* < 0.001), with an R^2^ of 0.87 (Figure 1).

### 3.3. Attending Pre-Adolescent Training

The significant correlations, including those for background variables, for the models used to assess the relationship between attending pre-adolescent training and the occurrence of a specific behavior problem are provided below. Results are grouped in subsections by behavior problem (i.e., partitioned by analytical model). Numerical results for all relationships, including insignificant correlations, are available in the data repository.

#### 3.3.1. Aggression

Male dogs were found to have 0.68 the odds of developing aggressive behavior (95% CI: 0.53–0.88; *p* = 0.003). Attending training before, or at, 6 months of age was associated with 0.71 the odds of developing aggressive behavior (95% CI: 0.53–0.97; *p* = 0.030).

#### 3.3.2. Compulsion

Male dogs were found to have 0.66 the odds of developing compulsive behavior (95% CI: 0.49–0.88; *p* = 0.006). Attending training at an age of 6 months or younger was associated with 0.64 the odds of developing a compulsive behavior (95% CI: 0.45–0.92; *p* = 0.015).

#### 3.3.3. Coprophagia

Neutered dogs were found to have 2.01 the odds of exhibiting coprophagia (95% CI 1.30–3.19; *p* = 0.002).

#### 3.3.4. Destructive Behavior

Dogs acquired at 12 weeks of age or younger were found to have 0.50 the odds of exhibiting destructive behavior (95% CI: 0.31–0.79; *p* = 0.003). Attending training at an age of 6 months or younger was associated with 0.60 the odds of exhibiting destructive behavior (95% CI: 0.37–0.96; *p* = 0.035).

#### 3.3.5. Escaping/Running Away

Neutered dogs were found to have 1.97 the odds of escaping/running away (95% CI: 1.12–3.69; *p* = 0.025).

#### 3.3.6. Excessive Barking

Attending training at an age of 6 months or younger was associated with 0.68 the odds of excessive barking (95% CI: 0.47–0.99; *p* = 0.043).

#### 3.3.7. Fear/Anxiety

Neutered dogs were found to have 3.10 the odds of fear/anxiety (95% CI: 2.05–4.72; *p* < 0.001). Dogs acquired at 12 weeks of age or younger were found to have 0.65 the odds of fear/anxiety (95% CI: 0.46–0.92; *p* = 0.016).

#### 3.3.8. House Soiling

Attending training at 6 months of age or younger was associated with 1.56 the odds of house soiling (95% CI: 1.08–2.27; *p* = 0.019).

#### 3.3.9. Mounting/Humping

Male dogs were found to have 0.37 the odds of mounting/humping (95% CI: 0.26–0.52; *p* < 0.001).

#### 3.3.10. Problematic Jumping

**Figure 2:**
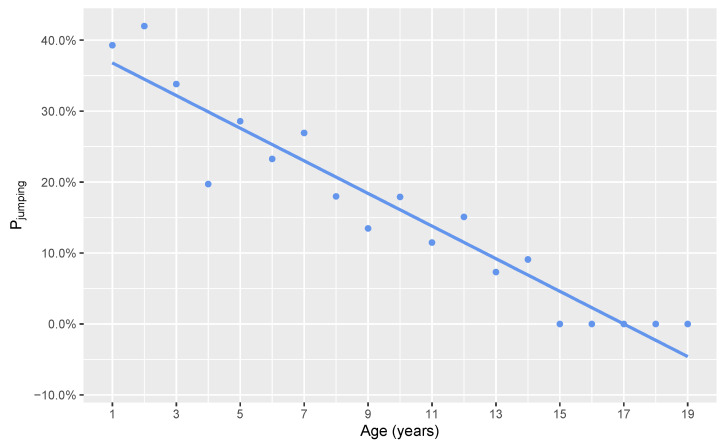
A smooth geometric means plot for the probability of exhibiting problematic jumping versus the current age of the dog in years.

An increase in age by 1 year was associated with 0.84 the odds of problematic jumping (95% CI: 0.80–0.88; *p* < 0.001) (Figure 2).

#### 3.3.11. Rolling in Repulsive Materials

Male dogs were found to have 1.53 the odds of rolling in repulsive materials (95% CI: 1.18–1.97; *p* = 0.001). Neutered dogs were found to have 1.72 the odds of rolling in repulsive materials (95% CI: 1.12–2.66; *p* = 0.014).

### 3.4. Pre-Adolescent Training Factors

Only one significant correlation, excluding correlations with background variables that were reported in the previous section, resulted from the models used to assess the relationship between pre-adolescent training factors and the occurrence of a specific behavior problem. The sole significant correlation was that dogs subjected to a reward-based training program had 0.52 the odds of developing aggressive behavior (95% CI: 0.28–0.96; *p* = 0.039). Numerical results for all relationships, including insignificant correlations, are available in the data repository.

## 4. Discussion

### 4.1. Principle Findings

We set out to investigate the potential effects of puppy training on the subsequent development of behavior problems in three different age groups up to 6 months of age and to compare positive reward-based versus punishment-based techniques. Even though most professionals advocate early training to coincide with the sensitive period of learning, we found no difference between the subsequent behavioral effects of puppy training when comparing across dogs that began puppy training at 1–3 months, 4 months, and 5–6 months of age. Instead, we found that puppy training at any age in the first 6 months of a puppy’s life was associated with reduced odds of adult dogs displaying aggression, compulsive behavior, excessive barking, and destructive behavior. It is possible that the reduced odds of certain behavior problems for dogs that attended puppy training is a result of owners seeking training specifically to address these problems that had already developed. However, a more likely explanation is that the odds of dogs developing the certain behavior problems were reduced *because* of the training they received as youngsters. In support of this contention, some of the problems noted are not usually seen in young puppies, are rarely problematic at this stage of life, and often take time to develop [23,24]. Destructive behavior in the form of puppies’ chewing behavior could be a reason for an owner seeking help in the form of puppy training. However, at this stage of life such behavior may not be interpreted as a serious condition in need of treatment. Likewise, excessive barking is more often an issue affecting older dogs and one that is more likely to be accepted as endearing in young puppies [25]. The behavior problems that we interpret as benefitting from puppy training were reported for adult dogs. The possible exception is house soiling, which occurs in all young dogs until they are house trained. In this case, the 1.54 odds of house soiling for dogs that attended puppy training may well be best explained by house soiling being the reason for puppies being brought for training. The finding that 78% of dogs in this study were reported to have had a house soiling problem makes this explanation most likely and indicates that the question may have been unclear and misinterpreted by owners.

The type of training employed was said to be largely reward-based, positive reinforcement training, though punitive devices were employed by 28% of owners claiming to have their puppies trained using reward-based methods. Reduced odds of aggression found in dogs attending puppy training class may be attributable to dog owners continuing to practice what they were taught in puppy training classes. In one study, dogs trained using reward-based techniques were better at performing a novel task and were more playful than those trained using punitive methods [5]. It seems reasonable to assume that the more trainable and playful dogs are less likely to be aggressive. In support of this notion, we found that puppy training based on reward-based methods substantially reduced the odds of aggression in adult dogs. The converse, that punishment-based methods increased the odds of aggression may explain this finding. In support of this contention, it has been found that more frequent use of punishment is associated with increased aggression and excitability [26]. Additionally, the use of punishment when training dogs has been found to be related to an increase in both fear and aggression [7].

### 4.2. Ancillary Findings

Males, most of which were neutered, had reduced odds of displaying aggressive behavior. That finding is not what has been found in other studies, where male dogs, neutered or otherwise, were found to be more aggressive than females [27,28]. Our contrary finding may be a result of some peculiarity in our sample, for example, that almost half of owners engaged their dog in puppy training during the first 6 months of their life. Alternatively, the multivariate statistical approach may have uncovered some novel aspect of canine aggression by demonstrating that aggression is truly more prevalent in a mostly neutered female dog population.

Male dogs in the entire population studied had reduced odds of displaying compulsive behavior. That result concurs with what has recently been reported for the human population, that is, that the prevalence of obsessive-compulsive disorder (OCD) in women is about 50% greater than in men [29]. Our finding, however, contrasts with our own previous findings [30] in which sex differences were not found for dogs reportedly showing compulsive behavioral signs and the findings of Overall and Dunham [31], in which male dogs were found to be more likely to exhibit compulsive disorders.

Neutered dogs of either sex were found to have just over twice the odds of exhibiting coprophagia. We found no sex difference in coprophagic dogs. However, a study of 1552 dogs by Hart et al. [32] found no difference in the distribution of coprophagy in sex or neuter status categories. Understanding motivational aspects of coprophagy continues to perplex researchers. Theories range from inherited ancestral wolf traits for keeping the den area free of intestinal parasites to ingesting feces as a result of poor environmental welfare and/or an inadequate diet [32,33].

We found that puppies acquired at 12 weeks of age or less had reduced odds of exhibiting destructive behavior. A possible explanation for this is that hyper-attachment is a key feature of canine separation anxiety [34], which may be less likely in dogs whose early life is not disrupted by late placement or adoption from shelters as juveniles or adults [35].

Neutered dogs in this study were almost twice as likely to be reported as “escaping” or “running away.” It is possible that this effect is influenced by the escape aspects of separation anxiety because separation anxiety is reportedly more prevalent in neutered male dogs [34,35,36,37]. Alternatively, dogs that often escaped or ran away may have been neutered to prevent the behavior. Roaming, akin to running away, is well known to be reduced by castration [38].

Excessive barking was negatively correlated with attending puppy training. The most plausible explanation for this result is that puppy training is helpful in most dogs in combating this annoying problem. The alternative explanation that attending puppy training increased the odds of a dog barking excessively seems less likely. Positive training methods, which were largely employed in this study, focus on rewarding silence as opposed to punishing barking. In our opinion, such training is more likely to result in the desired outcome than to make matters worse.

Neutered dogs had over three times the likelihood of developing into fearful adults. This finding concurs with a trend seen in our recent demographic behavioral study [30] and what others have found [39]. The fact that male and female hormones facilitate boldness and reduce anxiety might explain this otherwise baffling result [39,40].

Neither age of acquisition, sex, neuter status, nor attendance at puppy training made any impact on hyperactivity/overactivity. One possible explanation for the refractoriness of hyperactivity could be that attention deficit hyperactivity disorder (ADHD) type behavior in dogs may depend on a classical gene–environment interaction [41] and thus be relatively immune to training techniques.

We found that male dogs of either neuter status had significantly lower odds of mounting or humping. In concurrence, in a previous survey, we found that neutering reduced the prevalence of mounting/humping in males but left it the same or marginally increased in females [30]. Our current finding may be linked to the other finding that the odds of aggression were reduced, and aggression and mounting/humping behavior are biologically linked [42]. Neutering male dogs has been reported to decrease mounting behavior [38] though there is paucity of information about the behavioral effects of neutering females [43].

Our results indicate that the odds of a dog rolling in repulsive materials is influenced by the dog’s biological sex and neuter status. Male sex and neutering were found to increase the odds of rolling in repulsive materials. Paradoxically, in a previous study [30], we found that male dogs were less likely to engage in this behavior. In that study, the percentage of neutered dogs that rolled in repulsive materials was greater than that for unneutered dogs.

An interesting side finding of the present study was that the older a dog was the less likely it was to have attended puppy training sessions (Figure 1). This finding may be due to owners becoming more aware recently of the benefits of puppy training or a growing availability of puppy training classes.

### 4.3. Study Limitations

Limitations of this study include the fact that it required retrospective accounts of previous puppy training from dog owners and recollections can sometimes be unreliable. However, we believe that the large numbers of respondents in this study, many of whom had younger dogs and recent memories of their pet’s early history, in conjunction with the rigorous statistical approach, offset this conceivable weakness. Another possible limitation is that while we asked open-ended questions about the dogs’ behavior, trying to get an accurate description of what the dogs were doing, owner reporting is a poor substitute for a clinical diagnosis but is all that is available for studies based on distributed online surveys.

The use of binary outcomes to represent the presence of behavior problems did not account for the severity of the presenting behavior problems. As a topic of further study, collecting data suitable for modeling with ordinal or continuous outcomes could be used to examine the impact of training on behavior problem severity. Due to the fact that detailed information about owners and households was not collected and that owners were not asked to specify the number of dogs in their household, the analytical models did not account for these factors. The distributed questionnaire was intended to collect information about pre-adolescent training only. Therefore, the analytical models do not account for training at other dog life stages. In addition, information was not collected about acquisition source and dog breed, so we were unable to account for these environment and genetic factors in our analytical models.

## 5. Conclusions

Dogs that had attended pre-adolescent training were less likely to have aggression, compulsive behavior, destructive behavior, and excessive barking compared to the control group. Frequency of attendance, the age at which training was started (within pre-adolescence), and the training devices employed were not found to have a significant impact on the outcome. Positive reinforcement training was associated with a reduced likelihood of aggressive behavior.

## Data Availability

The data and source code for this analysis are available to the public via a Mendeley data set at http://dx.doi.org/10.17632/8zxcr7mdz2.1 (accessed on 1 March 2021).

## Supplementary Materials

The following are available online at https://www.mdpi.com/article/10.3390/ani11051298/s1. File S1: An annotated copy of the pre-adolescent training questionnaire used to collect data for this study.
