# Supplementary material for: An Investigation into the Impact of Pre-Adolescent Training on Canine Behavior
Source: Animals (Basel). 2021 Apr 30;11(5):1298. doi: 10.3390/ani11051298 (PMC8147152; doi:10.3390/ani11051298)
Supplement: Supplementary file 1 [file animals-11-01298-s001.zip › supplementary/s1_questionnaire.pdf]

Pre-Adolescent Training Questionnaire

Hosted by the Center for Canine Behavior Studies

Owner Questions

The owner questions serve to gather demographic information about dog owners and the email address (which serves as a unique identifier) is used to associate dog owner responses with the responses for their dog. These questions are asked once per dog owner. Required questions are indicated with an asterisk.

1. What is your email? * [email]
2. What is your postal code (zip code)? [string]
3. What is your gender? [choose 1]
    A. Female
    B. Male
4. What is your age? [integer]

Dog Questions

Questions 1 through 38 are asked for each dog. Survey branching logic is indicated in italic text with a green background. Required questions are indicated with an asterisk.

1. What is the name of the dog you would like to submit a response for? * [string]
2. Was <dog> acquired at 12 weeks or younger? * [choose 1]
    A. Yes
    B. No
    C. I don't know

If Q2 != Yes, then skip to Q5

3. Is <dog> currently at least 1 year old? * [choose 1]
    A. Yes
    B. No
    C. I don't know

If Q3 != Yes, then skip to Q5

4. How many years old is <dog>? * [integer]
5. What sex is <dog>? * [choose 1]
6. Is <dog> spayed or neutered? * [choose 1]
    A. Yes
    B. No
    C. I don't know
7. Did you take <dog> for puppy training when he/she was 6 months old or younger? * [boolean]

If Q7 != Yes, then skip to Q12

8. At what age(s) did you take <dog> for training? * [choose all that apply]

    A. 3 months or younger
    B. 4 months
    C. 5-6 months
    D. I don’t know
9. How many classes did you and <dog> attend? * [choose 1]
    A. 1-3 classes
    B. 4-6 classes
    C. 7-9 classes
    D. 10+ classes
    E. I don’t know
10. At puppy training classes, what training techniques were used? * [choose 1]
    A. Rewarding techniques (e.g., treats, praise, pets)
    B. Tough love techniques (e.g., yelling, bopping on the nose, swatting on the rump, alpha rolls (pinning on back until dog submits), use of aversive collars (e.g., shock, prong, choke), jerking on the leash, water spraying, scruffing)
    C. A combination of rewarding and tough love techniques
11. What restraining/training devices were employed? * [choose all that apply]
    A. Nylon slip collar
    B. Buckle collar
    C. Head halter (with nose band)
    D. Harness (around chest)
    E. Metal "choke" collar
    F. Prong collar
    G. Martingale collar
    H. Electric shock collar
    I. No devices were employed
    J. Other: __________
12. Is there at least one situation in which <dog> behaves aggressively? * [boolean]

If Q12 != Yes, then skip to Q14

13. Who or what has <dog> acted aggressively toward? * [choose all that apply]
    A. Familiar people in the home
    B. Familiar people away from the home
    C. Strangers visiting the home
    D. Strangers away from the home
    E. Familiar dogs in the home
    F. Familiar dogs away from the home
    G. Unfamiliar dogs in the home
    H. Unfamiliar dogs away from the home
    I. Animals other than dogs
    J. Veterinarians
    K. Groomers
    L. Trainers
    M. Other: __________
14. Is there at least one situation in which <dog> is fearful or anxious? * [boolean]

If Q14 != Yes, then skip to Q16

15. What sort of fears and/or anxiety has <dog> had? * [choose all that apply]
    A. Fear of crowds
    B. Fear of veterinary visits
    C. Fear of noises
    D. Fear of thunderstorms
    E. Fear of other dogs
    F. Fear of animals other than dogs
    G. Separation anxiety
    H. Travel anxiety
    I. Generalized anxiety
    J. Post-traumatic Stress Disorder (PTSD)
    K. Other: __________
16. Do you consider <dog> to have a problem with jumping up on people? * [boolean]

If Q16 != Yes, then skip to Q18

17. Who has <dog> jumped up on? * [choose all that apply]
    A. Owners
    B. Familiar people
    C. Strangers
    D. Other: __________
18. Does <dog> bark excessively? * [boolean]

If Q18 != Yes, then skip to Q20

19. When has <dog> excessively barked? * [choose all that apply]
    A. At triggers (inside)
    B. At triggers (outside)
    C. Owners are present
    D. Owners are away
    E. To get attention
    F. During car rides
    G. Other: __________
20. Has <dog> ever eaten feces? * [boolean]

If Q20 != Yes, then skip to Q22

21. What type of feces has <dog> eaten? * [choose all that apply]
    A. Their own feces
    B. Dog feces other than their own
    C. The feces of other animals
    D. Other: __________
22. Has <dog> ever had any repetitive behaviors? * [boolean]

If Q22 != Yes, then skip to Q24

23. What sort of repetitive behaviors have you seen with <dog>? * [choose all that apply]
    A. Licking of the wrist/hock
    B. Tail chasing
    C. Digging in the yard
    D. Spinning

    E. Nail biting
    F. Shadow/light chasing
    G. Sucking flank regions/blankets
    H. Running in geometric patterns
    I. Collecting/arranging objects
    J. Stone/rock chewing
    K. Fly snapping (when no flies are around)
    L. Other: __________
24. Have you ever had a problem with <dog> soiling in the house? * [boolean]

If Q24 != Yes, then skip to Q28

25. When has <dog> soiled in the house? * [choose all that apply]
    A. Owners are present
    B. Owners are away
    C. Excited/overwhelmed
    D. Other: __________
26. How has <dog> soiled in the house? * [choose all that apply]
    A. Feces
    B. Urine
    C. Both feces and urine
    D. Other: __________
27. Where has <dog> soiled in the house? * [choose all that apply]
    A. Specific locations
    B. Anywhere
    C. Other: __________
28. Has <dog> ever rolled in repulsive material? * [boolean]

If Q28 != Yes, then skip to Q30

29. In what repulsive material has <dog> rolled? * [choose all that apply]
    A. Urine
    B. Feces
    C. Garbage
    D. Dead stuff
    E. Other: __________
30. Do you consider <dog> to be overactive/hyperactive? * [boolean]

If Q30 != Yes, then skip to Q32

31. In what ways has <dog> been overactive/hyperactive? * [choose all that apply]
    A. Difficulty settling
    B. Constant moving/jumping
    C. Impulsiveness
    D. Easily distracted
    E. Other: __________
32. Has <dog> ever exhibited destructive behavior in the home (other than puppy chewing behavior)? * [boolean]

If Q32 != Yes, then skip to Q34

33. When has <dog> been destructive? * [choose all that apply]

    A. Owner is present
    B. Owner is away
    C. Other: __________

34. Has <dog> ever run away/escaped? * [boolean]

If Q4 != Yes, then skip to Q36

35. Which of the following describes how <dog> has run away/escaped? * [choose all that apply]
    A. Escapes from home
    B. Escaped when out
    C. Escapes from confinement
    D. Returns home after escape
    E. Other: __________
36. Have you ever had a problem with <dog> mounting/humping? * [boolean]

If Q36 != Yes, then skip to Q38

37. Who or what has <dog> mounted/humped? * [choose all that apply]
    A. People
    B. Familiar dogs
    C. Unfamiliar dogs
    D. Inanimate objects
    E. Other: __________
38. Do you have another dog you would like to complete the questionnaire for? * [boolean]
